# Supplementary material for: 3,5-Dimethyl-2,4,6-trimethoxychalcone Lessens Obesity and MAFLD in Leptin-Deficient ob/ob Mice
Source: Int J Mol Sci. 2024 Sep 11;25(18):9838. doi: 10.3390/ijms25189838 (PMC11432508; doi:10.3390/ijms25189838)
Supplement: Supplementary file 1 [file ijms-25-09838-s001.zip › ijms-3139529-supplementary.pdf]

	Control		TriMetChalc		Control *vs.* TriMetChalc
	**Mean**	**SEM**	**Mean**	**SEM**	***t*-test, *p* value**
**Total Ceramides**	**402.23**	**47.63**	**402.26**	**15.55**	**0.99**
L_Cerd18_1.16_0	19.34	1.14	19.86	1.03	0.74
L_Cerd18_1.16_1	215.16	36.57	237.71	10.84	0.56
L_Cerd18_1.18_0	9.70	0.65	8.86	0.38	0.28
L_Cerd18_1.18_1	1.35	0.25	1.28	0.17	0.83
L_Cerd18_1.20_0	3.94	0.25	3.71	0.12	0.43
L_Cerd18_1.22_0	18.06	1.41	15.56	0.7	0.14
L_Cerd18_1.24_0	58.98	4.51	50.49	2.47	0.13
L_Cerd18_1.24_1	75.18	5.47	64.31	3.96	0.13
L_Cerd18_1.26_0	0.32	0.03	0.28	0.01	0.29
L_Cerd18_1.26_1	0.17	0.01	0.17	0.01	0.94
**Total SM**	**699.95**	**58.46**	**616.85**	**20.43**	**0.22**
L_SM18_1.14_0	1.36	0.13	1.23	0.03	0.37
L_SM18_1.16_0	205.99	14.28	201.18	9.08	0.78
L_SM18_1.16_1	2.65	0.22	2.83	0.13	0.49
L_SM18_1.18_0	54.47	5.49	49.74	3.52	0.48
L_SM18_1.18_1	2.16	0.19	2.33	0.18	0.55
L_SM18_1.20_0	18.55	2.16	17.75	1.15	0.75
L_SM18_1.20_1	2.16	0.20	2.03	0.08	0.58
L_SM18_1.22_0	76.53	8.99	65.07	3.70	0.27
L_SM18_1.22_1	19.90	1.57	18.30	0.32	0.36
L_SM18_1.24_0	79.55	7.95	66.85	2.49	0.17
L_SM18_1.24_1	236.59	19.13	189.49	4.54	0.05

Table S1. Detailed ceramides and spingomyelin composition and quantification of liver extracts from vehicle (*n* = 8)- or TriMetChalc (65 mg/kg BW, *n* = 8)-treated *ob*/*ob* mice. Statistical differences were examined by Student's paired *t*-test. *p* values are reported in the last column. A *p* value < 0.05 denotes statistical significance between vehicle- and TriMetChalc-treated mice.
